# Aging as Loss of Biological Self-Correction: A Geroscience Framework for Longevity, Inflammaging, and Restored Resilience

**DOI:** 10.3390/geriatrics11050134

**Published:** 2026-09-16

**Authors:** David Bar-Or, Jason Williams, Daniel Paredes

**Affiliations:** 1 Trauma Research Department, Swedish Medical Center, Englewood, CO 80113, USA; 2 Knoebel Institute for Healthy Aging, University of Denver, Denver, CO 80208, USA

**Keywords:** aging, longevity, geroscience, healthspan, biological resilience, inflammaging, NAD+, senescence, senolytics, epigenetic clocks

## Abstract

Longevity science has shifted from the pursuit of lifespan extension toward preservation of healthspan, resilience, and functional independence. The hallmarks of aging provide a powerful descriptive structure, but they do not by themselves specify how diverse molecular defects translate into loss of adaptive function. We propose a complementary conceptual framework in which aging is viewed as progressive impairment of biological self-correction: the distributed capacity to sense consequential perturbations, scale an appropriate response, repair or remove damage, terminate the response, and recover or adaptively re-equilibrate while preserving future functional reserve. This framework is not intended to replace homeostasis, homeodynamics, allostasis, physiological reserve, or resilience; rather, it links these established concepts to the molecular machinery that generates dynamic recovery. Correction fidelity is proposed to be measured independently of the hallmarks using stress-response trajectories, including response latency, magnitude of deviation, recovery slope or half-time, integrated deviation, residual deficit, overshoot, biological cost, final functional state, and preservation of subsequent reserve. Importantly, successful recovery need not mean return to a fixed youthful baseline because aging may also shift the physiological setpoint toward a less favorable but actively defended equilibrium. We integrate evolutionary and biodemographic principles with inflammaging, nutrient sensing, NAD+ biology, senescence, autophagy, partial reprogramming, and resilience research, and propose falsifiable predictions for testing whether dynamic recovery adds information beyond chronological age, frailty, and static aging biomarkers.

## 1. Introduction

The field of longevity science is undergoing a conceptual transition. Earlier discussions frequently emphasized lifespan extension, whereas contemporary geroscience increasingly emphasizes healthspan, resilience, functional capacity, and delay of age-related disease clusters [1,2]. This transition is clinically important. A longer life accompanied by frailty, cognitive decline, sarcopenia, immune dysfunction, osteoarthritis, cardiovascular disease, and loss of independence does not represent successful longevity. The more meaningful aim is preservation of physiological reserve and the ability to recover after biological stress.

Aging is the major risk factor for most chronic diseases, including cardiovascular disease, dementia, cancer, diabetes, osteoarthritis, sarcopenia, chronic kidney disease, immune dysfunction, and frailty [1,2,3]. Geroscience emerged from the recognition that age-related diseases are not isolated events but downstream expressions of shared biological vulnerabilities [1,2]. This perspective suggests that interventions targeting fundamental aging biology may delay or attenuate multiple diseases simultaneously rather than treating each late-life disease separately.

The hallmarks of aging framework has been instrumental in organizing this field. The original hallmarks described nine interconnected processes: genomic instability, telomere attrition, epigenetic alterations, loss of proteostasis, deregulated nutrient sensing, mitochondrial dysfunction, cellular senescence, stem-cell exhaustion, and altered intercellular communication [4]. The expanded 2023 framework added disabled macroautophagy, chronic inflammation, and dysbiosis, emphasizing that aging is not only intracellular but also immunologic, microbial, systemic, and tissue-context dependent [5].

The hallmarks describe recurrent molecular and cellular changes during aging more clearly than they specify how those changes alter the organism’s ability to respond to perturbation. A complementary organizing principle may therefore be useful. We propose that aging can be viewed as progressive impairment of biological self-correction, while emphasizing that this construct overlaps with and builds upon established concepts of homeostasis, homeodynamics, allostasis, physiological reserve, robustness, and resilience [6,7,8,9]. The proposed contribution is not a new molecular pathway, but an integrative process model linking sensing, response scaling, repair or removal, signal termination, and subsequent functional recovery or adaptive re-equilibration.

Life is not simply chemical activity. It is organized chemistry that preserves itself by sensing deviation, repairing damage, regulating internal state, adapting to perturbation, storing information, and reproducing across time. DNA repair, proteostasis, autophagy, mitophagy, lysosomal clearance, antioxidant regulation, immune surveillance, inflammatory resolution, tissue remodeling, stem-cell renewal, and metabolic recalibration are not isolated biological functions. They are elements of a larger self-correcting architecture.

With age, this architecture may lose fidelity in more than one way. Responses can become delayed, incomplete, excessive, energetically costly, or misdirected; but the regulated state itself can also drift. An older system may therefore fail to recover around an appropriate setpoint, or it may efficiently return to a chronically inflammatory, metabolic, vascular, or autonomic equilibrium that has become maladaptive. Thus, aging should not be reduced to passive error accumulation or sluggish repair. It can also involve compensatory remodeling and active defense of less favorable physiological states.

This manuscript develops a conceptual geroscience framework in which longevity is related to preservation of adaptive regulatory capacity. It integrates evolutionary and biodemographic principles, the hallmarks of aging, inflammaging, NAD+ biology, nutrient sensing, cellular senescence, biological-age biomarkers, partial reprogramming, and physical-resilience research. The central empirical proposal is that biological age may be reflected not only by the state of the organism at rest but also by the shape, cost, and outcome of its trajectory through and after a defined perturbation.

Literature approach. This conceptual narrative review was developed through targeted PubMed/MEDLINE searches using combinations of terms related to aging/ageing, geroscience, resilience, homeostasis, homeodynamics, allostasis, biodemography, hallmarks of aging, inflammaging, inflammatory resolution, autophagy, lysosomal biology, NAD+, mTOR, AMPK, cellular senescence, epigenetic aging, partial reprogramming, physiological stress responses, exercise, and recovery. Priority was given to landmark primary studies, systematic reviews and meta-analyses, recent mechanistic reviews, and human translational studies. Reference lists of major reviews and primary studies were additionally screened. Because the objective was conceptual synthesis and hypothesis generation rather than exhaustive evidence enumeration or effect-size estimation, study selection was not systematic and no meta-analysis was performed.

## 2. Evolutionary Logic: Why Aging Exists Without Biological Purpose

Evolution has no purpose, foresight, or intention. Aging is therefore not best understood as a program designed to terminate life. Rather, aging emerges from the structure of natural selection under resource constraints. The force of natural selection is strongest for traits that affect early survival and reproductive contribution, but it declines for traits whose effects occur late in life. Harmful variants that impair early survival or reproduction are efficiently selected against, whereas variants that act only after reproductive contribution may persist [10,11,12].

Several classical theories explain this logic. Medawar proposed mutation accumulation, in which late-acting deleterious mutations are weakly removed by selection [10]. Williams proposed antagonistic pleiotropy, in which traits that are beneficial early in life may produce harm later [11]. Kirkwood’s disposable soma theory emphasized resource allocation: organisms invest finite energy in growth, reproduction, defense, and repair, but indefinite somatic preservation is rarely selected when ecological mortality risk is high [12].

These theories do not imply that evolution prefers reproduction over maintenance. They imply that organisms are shaped by differential persistence under constraints. In such systems, perfect repair is costly, cancer suppression conflicts with regeneration, immune activation conflicts with inflammatory damage, and growth-promoting pathways may support early fitness while impairing late-life maintenance.

This framework also helps explain why aging is variable among species. Species differ in extrinsic mortality, reproductive timing, ecological protection, and investment in somatic maintenance. Modern biodemography provides an important bridge between these evolutionary ideas and geroscience. Large-cohort studies in Mediterranean fruit flies demonstrated late-life mortality deceleration rather than a universal monotonic rise in mortality, and subsequent comparative biodemographic work documented striking heterogeneity in mortality and longevity trajectories within and across species [13,14,15]. These observations caution against treating aging as a single fixed clock and instead emphasize population heterogeneity, life-history architecture, ecological context, and the failure dynamics of complex biological systems.

In the self-correction framework, evolution preserves sufficient correction to maintain lineage continuity, but it does not necessarily preserve somatic self-correction indefinitely. The germline carries information across generations; the soma is a temporary vehicle whose maintenance is optimized only within evolutionary constraints.

## 3. Life as a Self-Correcting System

Living systems constantly resist disorder. They maintain boundaries, process energy, store information, adapt to environmental change, and repair internal damage. This requires continuous error detection and correction.

At the molecular level, DNA lesions are repaired, misfolded proteins are refolded or degraded, oxidized lipids are remodeled, and damaged organelles are removed. At the cellular level, autophagy, mitophagy, lysosomal degradation, unfolded protein responses, antioxidant defenses, and membrane repair maintain viability [4,5]. At the tissue level, inflammation, coagulation, angiogenesis, matrix remodeling, immune clearance, and stem-cell activation restore structure after injury [16,17]. At the organismal level, endocrine, neural, immune, vascular, and metabolic loops maintain temperature, glucose, blood pressure, oxygen delivery, acid-base balance, and host defense.

These systems are interdependent. NAD+ metabolism links energetic state with DNA repair, sirtuin activity, mitochondrial function, and inflammatory restraint [18]. mTOR integrates nutrient abundance with growth, protein synthesis, autophagy, and immune activation [19]. AMPK senses energetic stress and shifts cells toward catabolism, mitochondrial quality control, and adaptive restoration [20]. NF-kB coordinates injury and immune responses but can become chronically activated during inflammaging [16]. Phosphatases restrain kinase-driven signaling and help terminate inflammatory and stress responses. Specialized pro-resolving mediators actively terminate inflammation and restore tissue homeostasis [21].

Thus, biological self-correction is best treated as a process construct rather than a synonym for homeostasis or resilience (Table 1). Homeostasis describes regulation within acceptable ranges; allostasis and homeodynamics emphasize stability through adaptive change; physiological reserve describes capacity available before a stressor; and resilience describes the observed ability to resist or recover after a stressor [6,7,8,9]. In the present framework, biological self-correction refers to the distributed mechanisms that generate those responses, whereas correction fidelity refers to how appropriately, efficiently, and completely the process is executed.

Operationally, self-correction can be decomposed into five stages: (1) sensing a consequential perturbation; (2) selecting and scaling a response; (3) repairing, removing, clearing, or adapting to the disturbance; (4) terminating and resolving the response; and (5) restoring function or establishing an adaptive new equilibrium. Correction fidelity can then be approached through independently measured trajectory features such as response latency, proportionality, overshoot, recovery time, residual deviation, biological cost, and preservation of subsequent functional reserve.

## 4. Aging as Loss of Correction Fidelity

Aging is often described as damage accumulation, but damage alone does not fully explain the aging phenotype. Young organisms also experience damage yet frequently repair, remove, resolve, or compensate for it efficiently. We therefore distinguish four interacting levels rather than treating all hallmarks as equivalent causes: initiating perturbations and constraints; sensing and corrective machinery; adaptive or maladaptive intermediate states; and downstream organismal phenotypes such as reduced reserve, delayed recovery, frailty, and disability. Hallmarks can occupy different positions in this hierarchy and may function as both causes and consequences through feedback. They are therefore candidate mechanisms of impaired recovery, not evidence that ‘correction failure’ exists by definition.

### 4.1. Genomic Correction Failure

Genomic instability is a primary hallmark of aging [4,5]. DNA is continuously challenged by replication errors, reactive oxygen species, radiation, metabolic byproducts, and environmental exposures. In young cells, repair pathways preserve genomic integrity. With age, repair efficiency declines, mutations accumulate, chromatin organization changes, and cells may undergo apoptosis, senescence, dysfunction, or malignant transformation. Genomic damage therefore represents both injury and impaired correction.

### 4.2. Proteostatic Correction Failure

Proteins must fold properly, localize correctly, maintain solubility, and be degraded when damaged. Aging impairs chaperone systems, proteasomal degradation, autophagy, and lysosomal clearance [4,5]. Neurodegenerative proteinopathies illustrate the complexity of this problem but should not be reduced to single clearance nodes. Tau, α-synuclein, and TDP-43 pathology emerge from interacting abnormalities in protein folding, degradation, phosphorylation, phase behavior, intracellular trafficking, autophagy-lysosomal function, mitochondrial biology, RNA processing, inflammation, and cell-type-specific vulnerability [22]. Within the present framework, these proteins are examples of multifactorial proteostatic stress rather than proof of a unitary corrective defect.

### 4.3. Mitochondrial Correction Failure

Mitochondria are energetic organelles, stress sensors, and inflammatory regulators. Aging impairs mitochondrial biogenesis, mitophagy, electron transport efficiency, redox balance, and NAD+-dependent signaling [5,23]. Dysfunctional mitochondria generate reactive oxygen species, release danger-associated molecular signals, and amplify innate immune activation. Mitochondrial dysfunction therefore links metabolic failure to inflammaging [16].

### 4.4. Autophagic and Lysosomal Correction Failure

Disabled macroautophagy was added to the expanded hallmarks of aging because turnover of damaged cellular components is essential to cellular homeostasis [5]. Autophagy and lysosomal degradation remove dysfunctional organelles, misfolded proteins, intracellular pathogens, and damaged macromolecules. Age-related changes in flux are tissue- and context-dependent and may reflect both impaired degradation and compensatory responses to increased substrate burden. Accordingly, autophagic aging should not be viewed as a uniformly monotonic decline. The relevant question is whether flux, lysosomal capacity, and nutrient-responsive switching remain sufficient to meet stress and repair demands while preserving metabolic flexibility.

### 4.5. Tissue-Level Correction Failure

Tissue repair requires coordinated inflammation, vascular response, matrix remodeling, stem-cell activation, immune resolution, and restoration of architecture [16,17]. In aging, repair frequently shifts toward fibrosis, calcification, scarring, matrix stiffness, and loss of regenerative capacity. This is central to osteoarthritis, pulmonary fibrosis, chronic kidney disease, cardiac remodeling, and impaired wound healing.

Aging can therefore be conceptualized as altered dynamics after perturbation rather than simply failure to return to a fixed baseline. Three reference states should be distinguished: the individual’s prestressor state, a context-appropriate functional state that reflects age, disease, medications, training status, sleep and circadian phase, and a youthful or low-risk comparative state. Successful adaptation may involve re-equilibration rather than literal restoration of the prestressor value. Conversely, rapid return to an already pathological setpoint is not evidence of healthy recovery. Biological age may therefore be reflected by response magnitude, recovery kinetics, residual deficit, biological cost, and the quality of the final functional state [6,7,8,9].

## 5. Inflammaging: Correction Without Resolution

Inflammation is an adaptive response that detects injury or infection, recruits effector cells, clears danger, and initiates repair. Inflammaging, however, cannot be reduced to a single failure of inflammatory resolution. It arises from persistent endogenous and exogenous stimuli, cellular senescence and SASP signaling, mitochondrial dysfunction and danger-associated molecular patterns, dysbiosis and barrier dysfunction, immunosenescence, altered nutrient and metabolic signaling, clonal hematopoiesis, impaired autophagy, and age-related changes in tissue microenvironments [5,16,24,25]. Impaired termination and resolution may be an important component of this network, but not its universal cause.

Inflammaging emerges from several convergent mechanisms. Senescent cells secrete cytokines, chemokines, proteases, and growth factors through the senescence-associated secretory phenotype [24]. Mitochondrial dysfunction releases danger signals [5,16]. Impaired autophagy fails to clear damaged organelles and inflammatory triggers [5]. Dysbiosis and barrier dysfunction provide chronic immune stimulation [5]. Epigenetic drift alters immune-cell programming [4,5,25]. NAD+ decline weakens sirtuin-mediated restraint of inflammatory transcription [23]. Defective resolution pathways prevent return to homeostasis.

The phrase‘ correction without resolution’ is therefore used only as a heuristic for one aspect of inflammaging. Aging can alter both inflammatory initiation and inflammatory closure. In some individuals the dominant abnormality may be persistent stimulation; in others, inadequate efferocytosis, lipid-mediator switching, or signal termination may prolong the response. Moreover, a chronically elevated inflammatory state can become a defended physiological setpoint rather than a transient response that is merely slow to resolve. The framework therefore asks whether inflammatory responses are appropriately scaled, terminated, and integrated with tissue restoration, rather than assuming that all chronic inflammation reflects the same mechanism.

This concept has therapeutic implications. Broad anti-inflammatory suppression may reduce cytokine levels while impairing host defense, wound healing, or adaptive stress responses. A geroscience approach should seek to restore inflammatory resolution, tissue repair, and return-to-baseline dynamics rather than merely suppress inflammatory initiation.

## 6. NAD+, Redox Biology, and Metabolic Resilience

Nicotinamide adenine dinucleotide is a central node linking metabolism, DNA repair, mitochondrial function, sirtuin activity, PARP signaling, CD38 activity, immune regulation, and stress adaptation [23]. NAD+ levels decline with aging in many tissues, although the degree and clinical relevance vary by tissue, disease state, and method of measurement.

Evidence concerning NAD+ should be separated into association, mechanism, and clinical efficacy. NAD+ concentrations and NAD+-dependent processes change with age in multiple tissues, but the magnitude and clinical significance of these changes are heterogeneous. Mechanistic studies show that experimental manipulation of NAD+ metabolism can influence DNA repair, sirtuin activity, mitochondrial function, redox biology, CD38-related metabolism, and inflammatory signaling [18]. In humans, nicotinamide riboside and nicotinamide mononucleotide generally increase NAD+-related metabolites, demonstrating biochemical target engagement, yet functional, metabolic, vascular, and other healthy-aging outcomes remain inconsistent across trials [23]. Raising an NAD+ biomarker therefore cannot be equated with clinical rejuvenation, and NAD+ decline should not be presented as a universally causal mechanism of human aging.

This inconsistency is important. NAD+ restoration may not be a universal anti-aging therapy. It may be most relevant in biologically selected subgroups with demonstrable NAD+ depletion, mitochondrial dysfunction, inflammatory burden, frailty, or impaired recovery after stress. Future trials should stratify by baseline metabolic state, inflammatory profile, tissue function, and biological-age phenotype.

Within the self-correction framework, NAD+ should not be viewed simply as a youth molecule. It is a metabolic-control molecule that couples energy availability to repair, stress tolerance, and inflammatory restraint.

## 7. Nutrient Sensing: mTOR, AMPK, Insulin/IGF-1, and Autophagy

Nutrient sensing is inherently dynamic. mTOR exists in two major complexes with overlapping but non-identical functions. mTORC1 integrates amino acids, growth factors, oxygen, and energetic state to promote translation and anabolic growth through effectors such as S6 kinase and 4E-BP proteins while restraining autophagic initiation through ULK1 and coordinating lysosomal/autophagic programs through TFEB-family regulation [19]. mTORC2 is more closely linked to growth-factor signaling, AKT/SGK/PKC pathways, cytoskeletal organization, cell survival, and metabolic control. These complexes therefore should not be treated as a single longevity switch. Recent work on amino acid–mTORC1 signaling illustrates the context dependence of this pathway: Rab1A-mTORC1 signaling supports β-cell PDX1 function and insulin biology, while sustained nutrient-responsive activation can also participate in metabolic disease [26,27]. Dietary restriction engages mTOR together with AMPK, insulin/IGF-1 signaling, sirtuins, autophagy, mitochondrial remodeling, and stress-response programs, with outcomes modified by age, sex, genotype, nutrient composition, and timing [28].

Rapamycin is one of the most robust pharmacologic lifespan-extending interventions in model organisms. In genetically heterogeneous mice, late-life rapamycin extended median and maximal lifespan, providing evidence that pharmacologic modulation of nutrient sensing can affect mammalian longevity [29]. However, translation to humans is complex. mTOR signaling is necessary for wound healing, immune function, muscle adaptation, and tissue growth. Chronic or excessive inhibition may impair essential adaptive responses.

Metformin has been proposed as a geroscience intervention because of its long clinical history, metabolic effects, AMPK-related signaling, and observational associations with reduced age-related disease burden [30,31]. The Targeting Aging with Metformin concept was designed to test whether metformin can delay a cluster of age-related outcomes rather than a single disease endpoint [30]. Whether metformin improves longevity in non-diabetic humans remains unsettled, but its importance lies in the trial paradigm: aging biology can be targeted as a shared risk architecture.

The lesson is that nutrient-sensing pathways require modulation rather than simplistic activation or blockade. Acute and chronic effects can differ, and tissue requirements are not interchangeable. Longevity may depend on maintaining the capacity to switch appropriately between growth and maintenance programs: anabolic signaling when tissue growth, immune activation, or repair is required, and autophagic, catabolic, and quality-control programs when nutrient or energetic conditions favor maintenance. This dynamic flexibility is more consistent with adaptive regulation than with a permanently ‘low-mTOR’ state.

## 8. Cellular Senescence: Protective Arrest That Becomes Pathological

Cellular senescence is a stress response that prevents damaged cells from proliferating. It is important for tumor suppression, wound healing, tissue remodeling, and embryologic development. However, with age, senescent cells accumulate and contribute to chronic inflammation, matrix degradation, immune dysfunction, and tissue decline through the senescence-associated secretory phenotype [5,32,33].

Preclinical studies have shown that senescent cell burden can contribute to physical dysfunction and reduced survival, while senolytic approaches can improve physical function and lifespan in old mice [33]. Early human pilot data suggest that dasatinib plus quercetin can reduce senescent-cell markers in selected clinical contexts, such as diabetic kidney disease, but human evidence remains preliminary [34].

The therapeutic challenge is specificity. Senescence is not uniformly harmful. Removing senescent cells indiscriminately could impair wound healing, tissue remodeling, or cancer suppression. Senomorphic approaches that suppress harmful secretory signaling without eliminating cells may be useful in some contexts, whereas senolysis may be preferable when senescent burden is clearly pathogenic.

In the self-correction model, senescence is initially an adaptive correction to prevent malignant transformation. Aging occurs when the senescence program itself becomes insufficiently corrected by immune clearance and tissue remodeling.

## 9. Epigenetic Drift, Biological Clocks, and the Measurement Problem

Epigenetic alterations are central to aging biology [4,5]. DNA methylation patterns, histone modifications, chromatin accessibility, and transcriptional programs change with age. Epigenetic clocks use these changes to estimate biological age or risk of age-related outcomes. DNAm PhenoAge, for example, was developed to capture risks for mortality and diverse aging-related outcomes [35].

Aging clocks are powerful tools, but they must be interpreted cautiously. Some clocks estimate chronological age, others predict mortality risk, disease burden, immune state, or pace of aging. A reduction in clock age after intervention does not necessarily prove rejuvenation unless accompanied by improved function, resilience, disease risk, or recovery capacity. Recent critiques emphasize that many aging clocks learn correlations rather than causal drivers of aging [36].

For geroscience trials, biological-age measures should be combined with functional endpoints. The Biomarkers of Aging Consortium has emphasized the importance of terminology, validation, and clinically relevant use cases for aging biomarkers [37]. Useful endpoints may include gait speed, grip strength, cardiorespiratory fitness, cognitive performance, immune response to vaccination, inflammatory resolution kinetics, mitochondrial reserve, wound healing, frailty indices, and recovery after standardized stressors.

The most clinically useful aging biomarker will not simply estimate years; it will predict vulnerability, reversibility, and therapeutic response.

## 10. Partial Reprogramming and Epigenetic Rejuvenation

Partial reprogramming is among the most conceptually important but still predominantly preclinical approaches in longevity science. In mice, expression of Oct4, Sox2, and Klf4 restored youthful methylation features and improved retinal ganglion-cell function and vision-related outcomes [38]. In cultured human cells, maturation-phase transient reprogramming produced multi-omic rejuvenation while preserving aspects of cellular identity [39]. These findings demonstrate biological plasticity, but they do not establish safe organismal rejuvenation in humans.

The translational barriers are substantial. Excessive or poorly controlled reprogramming can promote dedifferentiation, loss of lineage identity, dysplasia, teratoma formation, or other neoplastic risk. Additional uncertainties include genomic and epigenomic stability, tissue-specific responses, delivery and targeting, duration and reversibility of factor expression, immunogenicity, durability of any rejuvenated phenotype, and the need for long-term cancer surveillance. Human-cell experiments and animal studies should therefore be clearly distinguished from clinical evidence, which remains absent for organismal rejuvenation.

Within the self-correction framework, partial reprogramming can be viewed cautiously as an attempt to modify epigenetic state and restore regulatory competence. The phrase ‘reset biological memory’ is therefore metaphorical rather than evidence that a complete or safe biological-age reset has been achieved. Any future clinical strategy would require precise dosing, tissue targeting, temporal control, safety switches, and prolonged surveillance.

## 11. Resolution Biology: The Missing Half of Inflammation in Longevity

Most aging research has focused on inflammatory activation, but less attention has been given to active resolution. Resolution is not passive decay of inflammation. It is a coordinated biochemical process involving specialized pro-resolving mediators, macrophage efferocytosis, neutrophil clearance, tissue repair, and restoration of homeostasis [16,17].

Aging may impair lipid mediator class switching, shifting tissues toward persistent prostaglandin and leukotriene signaling rather than resolvin-, protectin-, maresin-, and lipoxin-mediated resolution. This may be particularly relevant to osteoarthritis, neuroinflammation, vascular disease, trauma recovery, sepsis survivorship, and chronic wound healing.

From a longevity perspective, resolution capacity may be as important as inflammatory burden. A person with a strong inflammatory response that resolves efficiently may be biologically younger than a person with modest inflammation that persists. Therefore, future geroscience studies should measure not only cytokine concentrations, but also resolution kinetics, efferocytosis, specialized pro-resolving mediators, macrophage phenotype transition, and return-to-baseline dynamics.

Longevity requires the ability to finish repair.

## 12. Phosphorylation Drift and Failure of Signal Termination

Aging biology often emphasizes pathway activation: NF-kB activation, inflammasome priming, mTOR signaling, MAPK activity, and kinase-driven stress responses [5,16,29,30,31,32]. Equally important is failure of signal termination. Phosphatases such as PP2A restrain excessive phosphorylation and regulate pathways involving NF-kB, MAPK, AKT, tau [40,41,42], cytoskeletal dynamics [43], and inflammatory signaling [42].

Aging may involve phosphorylation drift, in which kinase activity is not adequately balanced by phosphatase-mediated resetting. This concept is relevant to neurodegeneration, cancer, osteoarthritis, metabolic disease, and chronic inflammation. In biological self-correction, turning off a response is as important as turning it on. Failed termination converts adaptive signaling into chronic pathology.

Although this area requires further development, it offers a useful extension of geroscience: aging is not only failure of repair, but failure of reset.

## 13. Cytoskeletal and Architectural Aging

Cellular architecture is an underemphasized dimension of aging. Microtubules, actin, intermediate filaments, and their post-translational modifications regulate intracellular transport, mitochondrial distribution, lysosomal positioning, immune synapse formation, mechanotransduction, and cell polarity. Aging alters cytoskeletal dynamics and may impair the spatial organization required for effective correction [4,5].

Microtubule acetylation, tubulin modifications, and emerging metabolic modifications such as lactylation may link redox state, lactate biology, inflammation, and intracellular transport [44]. This is relevant to neurodegeneration, osteoarthritis, immune aging, and tissue repair [45,46]. If cells cannot properly move mitochondria, lysosomes, vesicles, receptors, and signaling complexes, they cannot efficiently restore homeostasis.

Longevity science should therefore expand beyond genome, mitochondria, and inflammation to include maintenance of cellular architecture and intracellular logistics.

## 14. Dynamic Recovery as a Testable Aging Phenotype

The proposal that aging should be studied through recovery trajectories builds directly on established work in physical resilience rather than replacing it. Physical resilience has been defined as the ability to resist or recover from functional decline following a health stressor, and geriatric investigators have emphasized repeated post-stressor measurements, dynamical systems, and the distinction between robustness and recovery [7,8,9]. The present framework extends this literature by linking whole-person trajectories to candidate molecular components of sensing, repair, resolution, and re-equilibration.

A dynamic-recovery experiment requires a defined perturbation, repeated measurements, and an explicit reference state. Potential standardized or clinically defined stressors include exercise or cardiopulmonary challenge, vaccination, oral glucose or mixed-meal challenge, elective surgery, acute infection, and rehabilitation after injury. Candidate readouts include gait speed, Short Physical Performance Battery, grip strength, fatigability, cardiorespiratory performance, glucose-insulin dynamics, heart-rate variability, cortisol, inflammatory mediators, and selected metabolic or mitochondrial measures. No single stressor or biomarker is expected to represent whole-organism resilience.

Recovery should be quantified as a trajectory rather than a binary event. Candidate parameters include pre-stressor variability, maximum deviation, latency to peak response, recovery slope, time to partial or near-complete recovery, integrated deviation over time, residual deficit at a prespecified point, overshoot or oscillatory instability, biological cost, final functional state, and preservation of reserve for a subsequent challenge. Crucially, the final state may represent adaptive re-equilibration rather than literal return to the prestressor value, and a chronically unfavorable but actively defended setpoint should not be mistaken for healthy recovery [6,7,8].

This formulation produces falsifiable predictions. Dynamic recovery metrics should predict clinically relevant outcomes such as frailty progression, postoperative recovery, disability, or mortality independently of chronological age and at least some static biomarkers. Individuals with similar resting profiles may show different recovery trajectories. Interventions that improve adaptation should improve one or more trajectory parameters even when static clock measures change little. Different mechanisms may affect different trajectory components for example, mitochondrial dysfunction may increase energetic cost whereas impaired inflammatory termination may prolong recovery. The framework would be weakened if these measures prove irreproducible, add no information beyond established predictors, or fail to change when candidate corrective mechanisms are experimentally manipulated.

## 15. What Should Count as a Longevity Intervention? Evidence Hierarchy and Exercise

A true longevity intervention should not be defined solely by lifespan extension in model organisms or improvement in a molecular biomarker. Evidence should be ranked. At the strongest clinical level are interventions with established health benefits—physical activity, resistance exercise, smoking avoidance, blood-pressure and cardiometabolic control, vaccination, sleep preservation, and dietary quality [47,48,49]. A second category includes clinically established drugs with geroscience hypotheses but no proven anti-aging indication in healthy humans. A third includes investigational human geroscience strategies such as TOR-pathway modulation, NAD+ precursors, and senolytic or senomorphic approaches. Partial reprogramming and related high-potency rejuvenation strategies remain predominantly preclinical. This hierarchy prevents biochemical plausibility from being mistaken for demonstrated extension of human healthspan [23,31,32].

Physical activity deserves special emphasis because it is both a controlled physiological perturbation and an intervention that trains the response to perturbation. Acute exercise challenges energetic, redox, inflammatory, cardiovascular, mechanical, and metabolic regulation; repeated training improves cardiorespiratory and neuromuscular reserve, insulin sensitivity, mitochondrial capacity, vascular function, and stress adaptation. Resistance training is particularly relevant in older adults because preservation of muscle strength and power expands reserve available during subsequent illness or injury. Observational evidence and meta-analysis associate resistance training with lower all-cause and cause-specific mortality, although such data do not by themselves prove a direct anti-aging mechanism [50]. Exercise therefore provides the clearest established example of improving adaptive capacity without requiring the claim that a single molecular hallmark has been ‘reversed.’

This distinction is important. Longevity medicine should remain anchored to function, independence, disease delay, and resilience rather than supplement use or isolated biomarker shifts. Experimental geroprotective strategies are scientifically important, but their evidence level should be stated explicitly and should not be presented as equivalent to interventions with established clinical benefit.

## 16. Proposed Framework: Aging as Altered Adaptive Regulation

**Figure 1:**
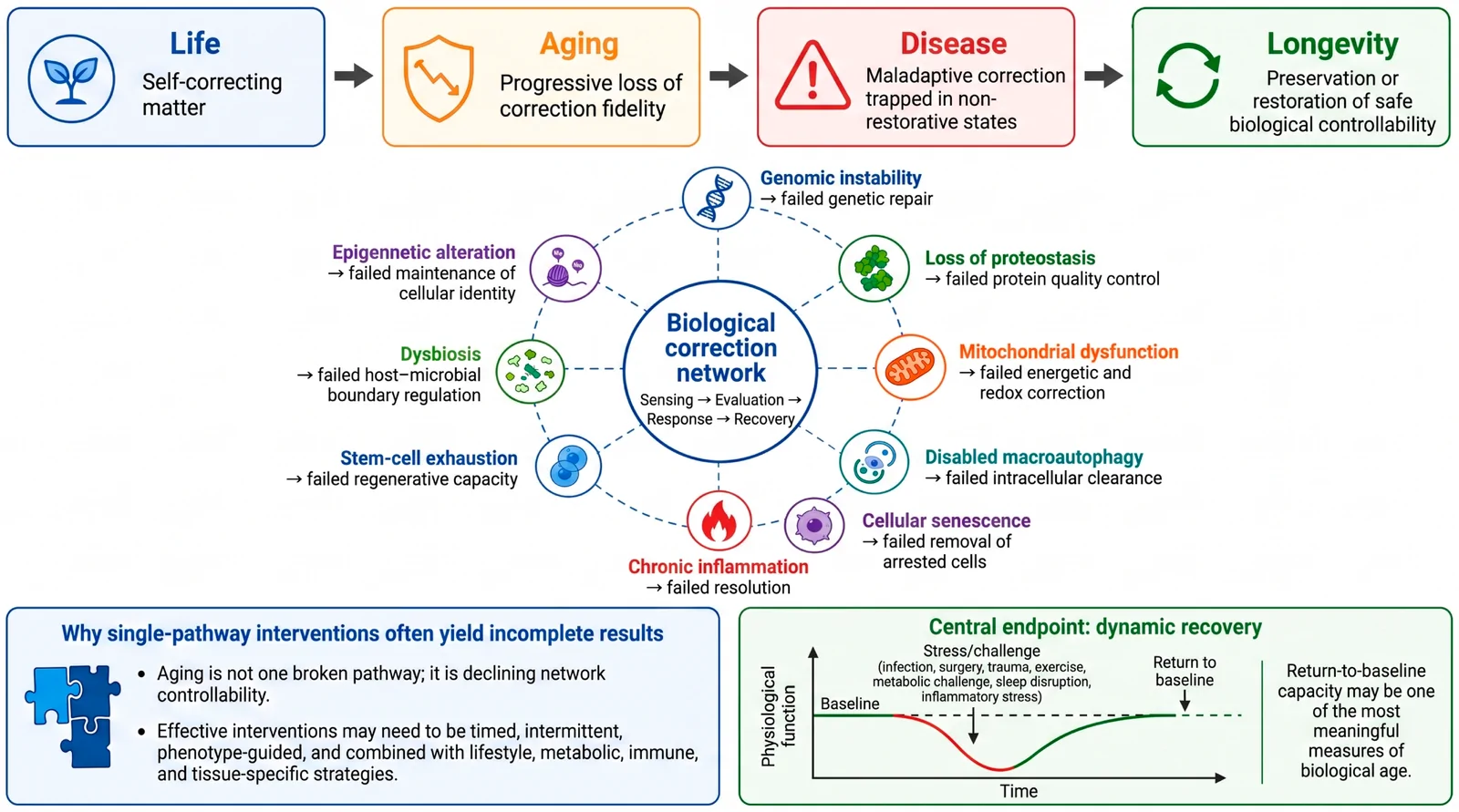
Biological correction network model of aging, disease, and longevity. The original schematic is retained because it visually summarizes the proposed framework: life as self-correcting matter, aging as progressive loss of correction fidelity, disease as maladaptive correction trapped in non-restorative states, and longevity as preservation or restoration of adaptive regulatory capacity. The central network maps major hallmarks of aging to candidate correction functions as a heuristic rather than as one-to-one causal assignments. The lower-left panel emphasizes why single-pathway interventions may produce incomplete effects. The lower-right panel depicts a simplified recovery trajectory after a stressor. The term ‘biological controllability’ in the schematic is used heuristically and corresponds to adaptive regulatory capacity in the revised text, not formal mathematical controllability. Likewise, the depicted baseline is a simplified reference state: successful recovery may involve return toward the prestressor state or adaptive re-equilibration, whereas aging may also establish a maladaptive but actively defended setpoint. Dynamic recovery should therefore be evaluated by response magnitude, recovery kinetics, biological cost, final functional state, and preservation of future reserve.

We propose the following summary model in Figure 1.

The model therefore reframes the hallmarks as candidate mechanisms within a larger hierarchy rather than as proof of correction failure. Genomic instability can increase the burden placed on repair systems; proteostatic and autophagic dysfunction can impair removal of damaged material; mitochondrial changes can affect energetic and redox capacity; senescence can be an initially adaptive arrest that becomes a source of chronic signaling; and inflammation can be both a response to injury and a driver of additional damage [5,16,17,24]. These relationships are reciprocal and tissue-specific. No one-to-one mapping between a hallmark and a single corrective failure is assumed.

The framework also clarifies why single-pathway interventions often produce incomplete results. Aging involves interacting mechanisms across different hierarchical levels, while some responses are compensatory or contextually beneficial. Successful interventions may therefore need to be timed, intermittent, phenotype-guided, and combined with behavioral, metabolic, immune, or tissue-specific strategies. We use the term adaptive regulatory capacity rather than formal ‘biological controllability’ because mathematical controllability has a specific meaning in systems theory. Where control terminology is used heuristically, the state is the multidimensional physiological condition, the perturbation is the stressor, inputs are endogenous responses or interventions, outputs are measurable molecular or functional variables, and feedback is information generated by the consequences of the response.

The central empirical endpoint is dynamic recovery, but not simple return to a fixed baseline. The clinically relevant question is how much function is disturbed, how rapidly and at what biological cost the system adapts, whether it terminates the response appropriately, what functional state is reached, and whether sufficient reserve remains for a future challenge. This approach links the molecular framework to established physical-resilience research and makes the model testable rather than circular [7,8,9].

## 17. Limitations and Future Directions

This framework has important limitations. It has not been prospectively validated as a unified model, overlaps substantially with homeodynamics, allostasis, physiological reserve, robustness, and resilience, and currently lacks a validated universal measure of correction fidelity. Causal ordering among aging mechanisms remains uncertain, and the same pathway can be adaptive in one tissue or time window and harmful in another. Aging is heterogeneous across organs and individuals, while prestressor states are influenced by chronic disease, medications, sleep, circadian timing, training status, behavior, and environment. Dynamic-recovery assays will therefore require stressor-specific and organ-specific validation rather than assumption of a single universal biological-age test.

Several priorities follow. Geroscience trials should combine biological-age biomarkers with functional and resilience endpoints, including repeated measures before and after clinically relevant or standardized stressors [7,8,9,35,36,37]. Reference states should be defined prospectively, preferably using repeated prestressor measures when feasible, and analyses should distinguish resistance to perturbation from recovery after perturbation. Studies should test whether trajectory parameters add prognostic or predictive information beyond chronological age, comorbidity, frailty, and resting biomarkers.

Interventions should also be stratified by biological state and evidence level. NAD+ precursors [23], TOR-modulating strategies [29], senolytics [33,34], metformin [30], and resolution-directed therapies are unlikely to benefit all individuals equally [31,32]. Mechanistic studies should determine which component of the trajectory is altered, response magnitude, repair, termination, recovery speed, final state, or biological cost, rather than assuming that improvement in a pathway marker represents generalized rejuvenation.

Inflammatory resolution and signal termination deserve particular attention, but neither should be treated as the sole explanation for inflammaging. Studies should measure both persistent inflammatory inputs and the kinetics of termination, efferocytosis, lipid-mediator switching, tissue remodeling, and movement toward either adaptive or maladaptive post-stressor states [16,17,21].

Combination approaches should be designed cautiously because aging is multifactorial but indiscriminate stacking of putative geroprotectors may produce antagonism or toxicity. Exercise and other established behavioral interventions should serve as comparators or foundations rather than being overshadowed by more experimental strategies.

Artificial intelligence and network methods may be useful in this context not as stand-alone aging clocks but as tools for integrating repeated multi-omic, physiologic, imaging, wearable, and functional measurements. Their most useful role would be to identify reproducible recovery phenotypes, test causal or mechanistic models, and determine whether specific interventions improve adaptive trajectories in defined biological states [36,37].

Finally, the framework should be judged by empirical performance. If dynamic recovery metrics are not reproducible, do not predict clinically meaningful outcomes, provide no information beyond established measures, or fail to respond to experimental manipulation of candidate mechanisms, then the proposed construct will require modification or rejection. The goal is therefore not to rename familiar aging biology, but to formulate a testable bridge between molecular maintenance systems and clinically meaningful recovery.

Longevity science should remain clinically grounded. The objective is not cosmetic reduction of a clock estimate but preservation of mobility, cognition, immune competence, tissue repair, independence, and the capacity to adapt safely to future stress.

## 18. Conclusions

Longevity science is moving from descriptive catalogues of age-associated change toward interventions that target shared mechanisms of functional decline. The present framework is offered as an integrative, testable hypothesis rather than a replacement for established theories of homeostasis, homeodynamics, allostasis, reserve, or resilience. Its central premise is that aging may be expressed not only in the resting state of a biological system but also in the quality of its response to perturbation.

Biological self-correction is defined here as the distributed capacity to sense consequential change, scale an appropriate response, repair or remove damage, terminate that response, and recover or adaptively re-equilibrate while preserving future reserve. Aging can impair this cycle through slower or more costly recovery, incomplete termination, reduced repair capacity, or drift toward maladaptive but actively defended setpoints. The hallmarks of aging are therefore treated as interacting candidate mechanisms within this hierarchy rather than as circular evidence that correction failure exists.

The practical implication is that longevity may be better characterized by dynamic recoverability than by static preservation alone. Future studies should test whether response magnitude, recovery kinetics, biological cost, final functional state, and preservation of reserve improve prediction of frailty, disability, therapeutic response, or mortality beyond chronological age and conventional biomarkers. Successful longevity interventions should ultimately preserve the capacity to recover, adapt, and remain functionally coherent across time.

## Figures and Tables

**Table 1:** Distinguishing related constructs used in the proposed framework.

Construct	Operational Meaning in This Framework
Homeostasis	Maintenance of physiological variables within an acceptable operating range.
Allostasis/homeodynamics	Adaptive regulation and stability through change; the operating state itself can shift with context.
Physiological reserve	Capacity available before a stressor or challenge.
Robustness/resistance	Ability to limit the magnitude of deviation during a perturbation.
Resilience/dynamic recovery	Observed ability to recover function after a perturbation.
Biological self-correction	Distributed processes of sensing, response scaling, repair/removal, termination/resolution, and adaptive restoration.
Correction fidelity	Performance of the correction cycle, assessed by latency, proportionality, recovery kinetics, residual deficit, biological cost, final state, and preserved future reserve.

## Data Availability

No new data were created or analyzed in this study. Data sharing is not applicable to this article.
